# Application of Portable Near-Infrared Spectroscopy for Quantitative Prediction of Protein Content in *Torreya grandis* Kernels Under Different States

**DOI:** 10.3390/foods14111847

**Published:** 2025-05-22

**Authors:** Yuqi Gu, Haosheng Zhong, Jianhua Wu, Kaixuan Li, Yu Huang, Huimin Fang, Muhammad Hassan, Lijian Yao, Chao Zhao

**Affiliations:** 1 College of Optical, Mechanical and Electrical Engineering, Zhejiang A&F University, Hangzhou 311300, China; guyuqi@zafu.edu.cn (Y.G.); 2023612021010@stu.zafu.edu.cn (K.L.); 15968131641@163.com (Y.H.); 20080094@zafu.edu.cn (L.Y.); 2 Zhoushan Special Equipment Inspection Research Institute, Zhoushan 316021, China; 13656800858@163.com; 3 Panzhihua Academy of Agriculture and Forestry Sciences, Panzhihua 617061, China; jhuawu2024@163.com; 4 School of Agricultural Engineering, Jiangsu University, Zhenjiang 212013, China; fanghuimin@ujs.edu.cn; 5 U.S.-Pakistan Center for Advanced Studies in Energy (USPCAS-E), National University of Sciences and Technology, Islamabad 44000, Pakistan; hassan@uspcase.nust.edu.pk

**Keywords:** *T. grandis* kernel, portable NIRS, protein quantification, rapid detection, PLSR

## Abstract

Protein content is a key quality indicator in nuts, influencing their color, taste, storage, and processing properties. Traditional methods for protein quantification, such as the Kjeldahl nitrogen method, are time-consuming and destructive, highlighting the need for rapid, convenient alternatives. This study explores the feasibility of using portable near-infrared spectroscopy (NIRS) for the quantitative prediction of protein content in *Torreya grandis* (*T. grandis*) kernels by comparing different sample states (with shell, without shell, and granules). Spectral data were acquired using a portable NIR spectrometer, and the protein content was determined via the Kjeldahl nitrogen method as a reference. Outlier detection was performed using principal component analysis combined with Mahalanobis distance (PCA-MD) and concentration residual analysis. Various spectral preprocessing techniques and partial least squares regression (PLSR) were applied to develop protein prediction models. The results demonstrated that portable NIRS could effectively predict protein content in *T. grandis* kernels, with the best performance being achieved using granulated samples. The optimized model (1Der-SNV-PLSR-G) significantly outperformed models based on whole kernels (with or without shell), with determination coefficients for the calibration set ($R_{c}^{2}$) and prediction set ($R_{p}^{2}$) of 0.92 and 0.86, respectively, indicating that the sample state critically influenced prediction accuracy. This study confirmed the potential of portable NIRS as a rapid and convenient tool for protein quantification in nuts, offering a practical alternative to conventional methods. The findings also suggested its broader applicability for quality assessment in other nuts and food products, contributing to advancements in food science and agricultural technology.

## 1. Introduction

With the improvement in people’s living standards, there is an increasing concern about the health and safety of food [1,2]. *T. grandis* kernels are a kind of high-quality nut with both medicinal and edible properties [3]. They are oval or oblate in shape, with a brown outer shell; are covered by a thin, hard, dark brown or black pseudotesta; have a white or pale yellow inner kernel; and have a soft texture and sweet flavor [4]. The main cultivation zones for *T. grandis* kernels are predominantly found in several mountainous regions of eastern and southern China, including Zhejiang’s Kuaiji and Tianmu ranges, Anhui’s Huangshan district, Fujian’s southern highlands, northeastern Jiangxi, and the western mountain areas of Hunan Province [3,5]. Protein is a major nutrient in nuts, and its content significantly influences their color, taste, storage, and processing, which makes it a crucial indicator for evaluating the quality of nuts [6,7]. Several analytical techniques are routinely used to measure protein content, such as the Kjeldahl nitrogen analysis method, the biuret colorimetric assay, the dye-binding technique, and ultraviolet (UV) absorption spectroscopy [8]. However, these traditional chemical detection methods are time-consuming, costly, and environmentally polluting, requiring complex sample preparation that involves destructive crushing and the use of potentially hazardous reagents [9,10]. Additionally, chemical analysis is only suitable for sampling and is not applicable for large-scale testing in food trading. Therefore, it is necessary to develop an efficient, rapid method for determining the protein content in *T. grandis* kernels.

Currently, a variety of advanced detection technologies are being applied for the analysis of food components and quality, such as NIRS [11,12], hyperspectral imaging technology [13,14,15], laser-induced breakdown spectroscopy [16,17], and Raman spectroscopy [18]. Simultaneously, these detection technologies are being integrated with deep learning techniques to achieve rapid and accurate determination of detection targets [19,20,21]. Some studies have focused on the relationship between food production and the sustainable development of the environment and humanity [22,23,24]. NIRS technology utilizes the optical response characteristics of organic compounds within the near-infrared spectral range for analysis. It offers numerous advantages, including rapid analysis, no pollution, and no need for complex preprocessing [25]. Additionally, it enables analysis without damaging the structure of the sample itself [26,27,28]. This technology can be applied to determine the protein content in various types of nuts [7]. Qiu et al. employed NIRS technology to quantitatively detect the protein content in northeastern pine nuts. They established quantitative analysis models for both pine kernels with and without shells using the partial least squares (PLS) method. The determination coefficients of the calibration sets ($R_{c}^{2}$) for the protein models of pine kernels with and without shells were 0.91 and 0.94, respectively, while the root mean square errors (RMSEs) were 0.67 and 0.58, respectively, indicating the good predictive performance of the models [29]. Similarly, Hu et al. collected NIR spectrum data for chestnuts both with and without shells, developing predictive models for their protein content. The results showed that the determination coefficients ($R_{c}^{2}$) of the models established from the spectrum data of both states were above 0.87. The model based on the spectrum data of chestnut kernels (shelled chestnuts) demonstrated better predictive performance, with $R_{c}^{2}$ and $R_{p}^{2}$ values of 0.91 and 0.80, respectively [30]. Yi et al. developed NIRS prediction models to determine the moisture, protein, and fat contents in walnuts. The results demonstrated that the determination coefficients for these three components were all above 0.96 [31]. Shi et al. investigated the capability of NIRS for quantitatively detecting the amino acid and crude protein content in soybeans, exploring the effects of grinding particle size and fat content on the predictive performance of the NIRS models. The results showed that the determination coefficients for the NIRS models for crude protein and amino acids met the required standards ($R_{c}^{2}$ = 0.81–0.95). Additionally, grinding and lipid extraction were found to improve the accuracy of the prediction models [32]. Tang et al. established NIRS models for predicting protein and fat content in hickory nuts. The $R_{p}^{2}$ value of the protein model was greater than 0.85, and the root mean square error of the validation set was less than 0.05 [33]. Previous studies have successfully demonstrated the application of NIRS for protein quantification in various types of nuts. These studies have also established the theoretical foundation for exploring the NIRS quantitative prediction models of nuts in different states (in-shell, shelled, and kernels). However, these analyses were exclusively conducted using benchtop NIR spectrometers, which require (1) controlled laboratory conditions, (2) complex sample preparation, including the adjustment of sample physical state (moisture and temperature conditioning), the control of sample chemical stability (homogenization and particle size control), special cleaning methods, and (3) professional operation. These limitations fundamentally restrict their potential for field applications or rapid quality assessment in production settings. Building upon these foundational studies, but addressing their practical limitations, our study introduced two key methodological innovations. First, we employed portable NIRS technology that enabled on-site spectral acquisition without laboratory constraints. Second, we systematically investigated the portable NIR spectrum acquisition from *T. grandis* kernels in three distinct physical states (with/without shell and granules). Then, we explored the feasibility of developing protein prediction models using spectra obtained from each processing state, which represented a novel approach in nut quality analysis.

Based on the aforementioned information, there is limited research on NIRS for protein prediction in *T. grandis* kernels, and no studies have systematically evaluated the impact of kernel state (with/without shells and granules) on model performance. This gap hinders the development of reliable online detection systems for industrial applications. Therefore, this study aimed to (1) investigate the feasibility of using portable NIRS for rapid protein quantification in *T. grandis* kernels; (2) evaluate how different kernel states affect spectral data and model accuracy; and (3) develop optimized chemometric models to enable non-contact, high-throughput protein analysis. The findings could provide methodological guidance for real-time quality monitoring in nut processing, supporting the advancement of smart agriculture and precision food industry technologies.

## 2. Materials and Methods

### 2.1. Experimental Materials

**Figure 1:**
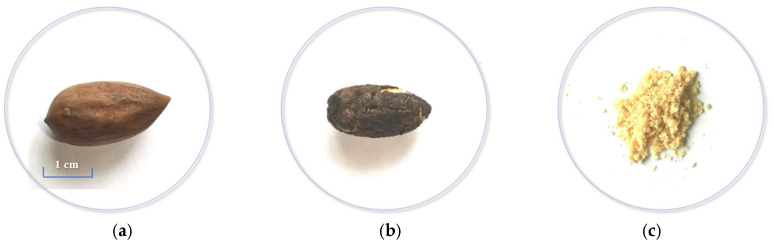
Three states of the *T. grandis* kernel. (**a**) *T. grandis* kernel with shell; (**b**) *T. grandis* kernel without shell; (**c**) *T. grandis* kernel granules.

The *T. grandis* kernels used in this study were sourced from smallholder farms in Zhanao Village (Jidong Town, Zhuji City, Zhejiang Province, China) and were harvested in September 2021. All samples were derived from fully mature *T. grandis* kernels with a growth cycle exceeding 30 months (from flowering to harvest). To explore the protein content of *T. grandis* kernels, portable NIR spectrum data were collected from three different states: (a) *T. grandis* kernels with shells, (b) without shells, and (c) kernel granules (as shown in Figure 1). We aim to explore the differences between the protein prediction models built based on portable NIR spectra from three different states and to determine whether the protein content of *T. grandis* kernels could be rapidly determined directly from the spectrum information of the shell in a non-destructive way. The same kernels were measured in their shelled form, their deshelled form, and then ground into granules. A sequential preparation protocol was implemented for comparative spectral analysis. Firstly, *T. grandis* kernels with shells were initially scanned using the portable NIR spectrometer. Secondly, each *T. grandis* kernel was carefully cracked using a laboratory hammer to preserve kernel integrity in relation to the pseudotesta (the dark protective layer surrounding the kernel, as shown in Figure 1b). *T. grandis* kernels without shells but with pseudotesta were scanned using the same portable NIR spectrometer. Lastly, the same kernel was ground using an agate mortar and then sieved through a 50-mesh sieve (particle size < 300 μm) to ensure uniformity. *T. grandis* kernel granules were scanned using the same portable NIR spectrometer. This paired-sample design ensured direct comparability of spectral data across all three states (*T. grandis* kernels with/without shells and granules) from identical biological samples. Particle size standardization was rigorously maintained through controlled grinding duration (15 min), uniform sieving protocol, and microscopic verification of granule morphology. The Kjeldahl nitrogen method measurements were performed on aliquots from the same homogenized granulated samples to maintain methodological consistency.

### 2.2. Portable NIR Spectrum Collection of T. grandis Kernels

The portable NIR spectra were acquired using a Smart Eye 1700 portable spectrometer (Huoyanjinjing Co., Ltd., Hangzhou, China) operated in the 1000–1650 nm spectral range with 1 nm resolution. The instrument was equipped with a dual-integrated vacuum tungsten lamp (NVC Lighting Co., Ltd., Guangzhou, Guangdong province, China) and a 128-element InGaAs array detector. All spectral measurements were conducted under controlled environmental conditions (25 ± 1 °C and 55% relative humidity). Prior to data collection, the spectrometer underwent a 30 min warm-up period to stabilize the system. For spectral acquisition, diffuse reflectance measurements were performed using a Spectralon white reference standard for background correction. The measurement parameters were set as follows: 50 accumulated scans per spectrum, 12.7 ms integration time, and 8 cm^−1^ spectral resolution [5]. Spectra were collected in the order of *T. grandis* kernels with shells, without shells, and granules. Each sample was placed in the sampling window area of the spectrometer, ensuring that the light source vertically illuminated the sample. After scanning, the *T. grandis* kernel granules were promptly placed in sample bags and the air was extracted from the bags, before being labeled with serial numbers and stored in a refrigerator for subsequent physicochemical analysis.

### 2.3. Determination of Protein Content in T. grandis Kernels

The Kjeldahl nitrogen method was selected as the reference method for protein content determination in *T. grandis* kernels because it is a well-established, standardized, and internationally recognized technique for total protein quantification in food and agricultural products. The specific operation steps were carried out in accordance with the Chinese National Food Safety Standard GB 5009.5-2016 “Determination of Protein content in Foods” [34]. The experimental instruments used include the KDN-04A Kjeldahl Nitrogen Analyzer (Lvbo Instruments Co., Ltd., Hangzhou, Zhejiang province, China) and the KDN-08C Digestion Furnace (Lvbo Instruments Co., Ltd., Zhejiang province, Hangzhou, China). The digestion reagents included copper sulfate (CuSO_4_), potassium sulfate (K_2_SO_4_), and sulfuric acid (H_2_SO_4_). The reagents used were 2% boric acid (H_3_BO_3_) solution (m/v), 40% sodium hydroxide (NaOH) solution (m/v), and 0.05 mol/L hydrochloric acid (HCl) standard solution. The mixed indicator solution was prepared by dissolving methyl red in ethanol to form a 0.1% ethanol solution, as well as by dissolving bromocresol green in ethanol to form a 0.5% ethanol solution, followed by mixing the two solutions in equal volumes. All chemicals were purchased from Sinopharm Chemical Reagent Co., Ltd. (Beijing, China) and were of analytical grade. The formula for calculating protein content is as follows:

(1) $$W=\frac{(v_{1}-v_{2})\times N\times 0.014\times F}{m}\times 100$$

where W is the mass fraction of protein content (%); *v*_1_ is the standard liquid consumption for blank titration (mL); *v*_2_ is the standard liquid volume consumed by reagent titration (mL); N is the concentration of hydrochloric acid standard solution (M); 0.014 is the millimolar mass of nitrogen; F is the protein coefficient; and m is the sample mass (g).

### 2.4. Principal Component Analysis (PCA) of T. grandis Kernels’ Portable NIR Spectra

The original portable NIR spectrum contains the complete spectral information of the samples, typically comprising thousands of wavelength points, resulting in high dimensionality. The high dimensionality of the spectral data can affect the speed of data analysis and the accuracy of model construction, so it is often necessary to reduce the dimensions of high-dimensional data [35]. Principal component analysis (PCA) is a commonly used method for the dimensionality reduction of high-dimensional data. By selecting principal components with the maximum variance, it is possible to filter out variables that best represent the characteristics of the original data, thereby eliminating redundancy and noise in the spectral data [36].

### 2.5. Elimination of Outlier Samples

In portable NIR spectroscopy, outlier detection was critical for ensuring model robustness. Outliers were classified as (1) spectral outliers (caused by instrumental errors or improper sampling techniques) and (2) chemical outliers (resulting from atypical protein content or reference method variability) [5,37]. Our outlier screening protocol employed a two-stage process. Spectral outliers were identified using PCA combined with Mahalanobis distance (PCA-MD). MD is a metric used to measure the similarity or difference between two samples. The MD of the samples was calculated and samples with excessively large MD were removed, thereby improving the accuracy of the model. The threshold of MD was empirically determined as 3× the median absolute deviation from the spectral PCA score cluster centroid [38]. Concentration residuals referred to the absolute error between the actual values determined by physicochemical experiments and the predicted values from the portable NIR spectrum quantitative model. Under normal conditions, concentration residuals were close to zero, indicating that the model’s predictions align with the true values. The concentration residuals method selected different thresholds based on this principle to eliminate samples with excessively large concentration residuals. In this study, samples were flagged as chemical outliers when the absolute difference between predicted and measured protein content exceeded 0.4%.

### 2.6. Sample Set Division of T. grandis Kernels’ Portable NIR Spectra

After removing outlier samples, the samples were divided into a calibration set and a prediction set at a ratio of 3:1 according to the X-Y distance metric (SPXY method). Here, the variable X represented the three states of portable NIR spectra of *T. grandis* kernel, and the variable Y represented the measured chemical data. By simultaneously calculating the distances between samples based on both X and Y variables, the SPXY method maximized the characterization of interactions and correlations within the sample distribution.

### 2.7. Establishment of a Quantitative Prediction Model for Determining Protein Content in T. grandis Kernels

When establishing a quantitative prediction model for determining protein content in *T. grandis* kernels, the baseline drift and light scattering noise of the original portable NIR spectrum can cause discrepancies between measured values and true values; therefore, it is essential to employ preprocessing methods to eliminate redundancy and noise in the spectrum [7]. The preprocessing methods adopted in this study include Savitzky–Golay smoothing (SG), normalization (Normalize), multiplicative scatter correction (MSC), standard normal variate (SNV), first derivative (1Der), second derivative (2Der), and a combination of two preprocessing methods. Among these, SG Smoothing reduced high-frequency noise (e.g., instrument noise, sample heterogeneity) while preserving spectral peak shapes. Normalize compensated for intensity variations caused by differences in sample concentration, thickness, or path length, ensuring spectra were on a comparable scale. MSC corrected for multiplicative scatter effects caused by particle size differences and surface scattering, common in powdered or irregularly shaped samples. SNV served a comparable purpose to MSC, but did not require a reference spectrum and processed each spectrum independently. Derivative removed baseline offsets and enhanced the resolution of overlapping peaks. These methods were screened to determine the most suitable preprocessing method for improving the accuracy of the model. Partial least squares regression (PLSR) is a statistical method used for regression analysis, and the model built by PLSR can explain the relationship between dependent variables and multiple independent variables. We used the “one standard error rule” to determine optimal latent variables (LVs) by identifying the LV number minimizing root mean square error (RMSE) and selecting the simplest model (fewest LVs) within one standard error of this minimum. PLSR combines the characteristics of PCA and multiple linear regression, making it particularly suitable for handling situations where independent variables exhibit high collinearity. The PLSR models were implemented using a full-spectrum approach that inherently incorporated all latent variables without manual selection. This method leveraged the entire spectral dataset to preserve covariance structures between predictors and responses, thereby eliminating the need for traditional latent variable optimization steps.

### 2.8. Evaluation of the Quantitative Prediction Model for Determining Protein Content in T. grandis Kernels

The accuracy of the quantitative prediction model for determining protein content in *T. grandis* kernels is evaluated by the determination coefficient ($R_{c}^{2}$) and root mean square error (RMSEC) of the calibration set. The $R_{c}^{2}$ value, which describes the degree of model fitting to the sample data, is calculated using Equation (2), while the RMSEC is calculated using Equation (3).

(2) $$R_{c}^{2}=1-\frac{\sum_{i=1}^{n_{1}}{\left(p_{i}-{\hat{p}}_{i}\right)}^{2}}{\sum_{i=1}^{n_{1}}{\left(p_{i}-\bar{p}\right)}^{2}}$$

(3) $$RMSEC=\sqrt{\frac{\sum_{i=1}^{n_{1}}{\left(p_{i}-{\hat{p}}_{i}\right)}^{2}}{n_{1}}}$$

where $p_{i}$ is the measured protein content of sample *i*; ${\hat{p}}_{i}$ is the predicted protein content of sample *i*; $\bar{p}$ is the mean measured protein content; and $n_{1}$ is the sample number of the calibration set.

The predictive ability of the model is assessed using the determination coefficient ($R_{p}^{2}$) and root mean square error (RMSEP) of the prediction set. Here, the $R_{p}^{2}$ value is used to test the model’s predictive performance, and is calculated using Equation (4), while the RMSEP is calculated using Equation (5).

(4) $$R_{p}^{2}=1-\frac{\sum_{j=1}^{n_{2}}{\left(p_{j}-{\hat{p}}_{j}\right)}^{2}}{\sum_{j=1}^{n_{1}}{\left(p_{j}-\bar{p}\right)}^{2}}$$

(5) $$RMSEP=\sqrt{\frac{\sum_{j=1}^{n_{2}}{\left(p_{j}-{\hat{p}}_{j}\right)}^{2}}{n_{2}}}$$

where $p_{j}$ is the measured protein content of sample *j*; ${\hat{p}}_{j}$ is the predicted protein content of sample *j*; and $n_{2}$ is the sample number of the prediction set. The higher the $R_{c}^{2}$ and $R_{p}^{2}$ values, and the lower the RMSEC and RMSEP values, the better the regression fitting effect of the model [39].

The ratio of performance to deviation (RPD) and the ratio of error range (RER) are employed to evaluate the quantitative prediction model. The RPD is calculated using Equation (6), while the RER is calculated using Equation (7).

(6) $$RPD=\frac{SD}{RMSEP}$$

(7) $$RER=\frac{\sqrt{\frac{\sum_{j=1}^{n_{2}}{\left({\hat{p}}_{j}-\bar{\hat{p}}\right)}^{2}}{n_{2}}}}{\sqrt{\frac{\sum_{j=1}^{n_{2}}{\left(p_{j}-\bar{p}\right)}^{2}}{n_{2}}}}$$

where $\bar{\hat{p}}$ is the mean predicted protein content of the prediction set.

## 3. Results and Discussion

### 3.1. Determination Results of Protein Content in T. grandis Kernels

The protein content in *T. grandis* kernels, determined using the Kjeldahl nitrogen method, is shown in Table 1. The analysis of protein content in *T. grandis* kernel samples revealed a broad distribution that encompassed and extended beyond the typical range (7.70–11.50%) reported in previous studies [40]. This wide variability in protein content in our samples enhanced the statistical robustness of our dataset, as it represented the natural diversity encountered in practical applications. Such comprehensive coverage of protein content ensured that the developed model would have greater predictive reliability. The substantial sample variation supported the model’s potential for accurate protein content prediction in real-world scenarios.

### 3.2. Portable NIR Spectrum Analysis of T. grandis Kernels

**Figure 2:**
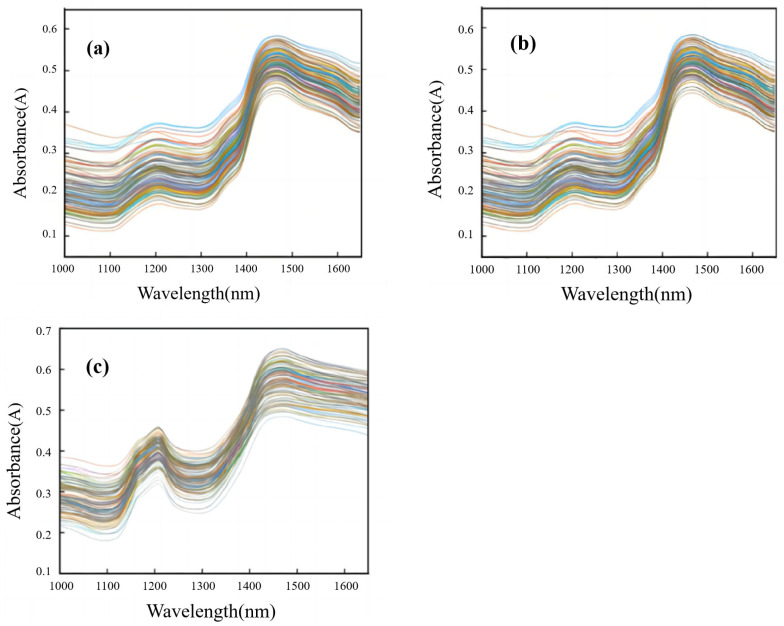
Portable NIR spectra of *T. grandis* kernels under different states. (**a**) *T. grandis* kernels with shells, (**b**) *T. grandis* kernels without shells, and (**c**) *T. grandis* kernel granules.

Figure 2 shows the original portable NIR spectra of *T. grandis* kernels under different states— *T. grandis* kernels with shells, *T. grandis* kernels without shells, and *T. grandis* kernel granules. According to previous studies, the characteristic functional groups of proteins mainly include –NH_2_, –NH, and –COOH in the wavelength range of 1000–1650 nm, and the peaks of protein absorption are primarily located at 1000–1200 nm and 1420–1520 nm [41]. As shown in Figure 2, there were two distinct absorption peaks in the portable NIR spectra of *T. grandis* kernels at 1200 nm and 1450 nm. The peak at 1200 nm corresponded to the stretching vibration of the carbon–hydrogen bond (C–H) in proteins, while the peak at 1450 nm corresponded to the stretching vibration of the amide bond (N–H) in proteins. Overall, the portable NIR spectrum trends in *T. grandis* kernels under three different states were consistent. However, the absorbance of *T. grandis* kernels without shells was higher than that of those with shells, while the absorbance of *T. grandis* kernel granules was higher than that of kernels without shells. In conclusion, the portable NIR spectrum absorption peaks of *T. grandis* kernels under the three states (with shells, without shells, and in the form of granules) aligned well with the wavelength ranges of the protein absorption peaks reported in the literature, and the collected spectra provided the necessary information for modeling.

### 3.3. Portable NIR Spectrum PCA of T. grandis Kernels

**Figure 3:**
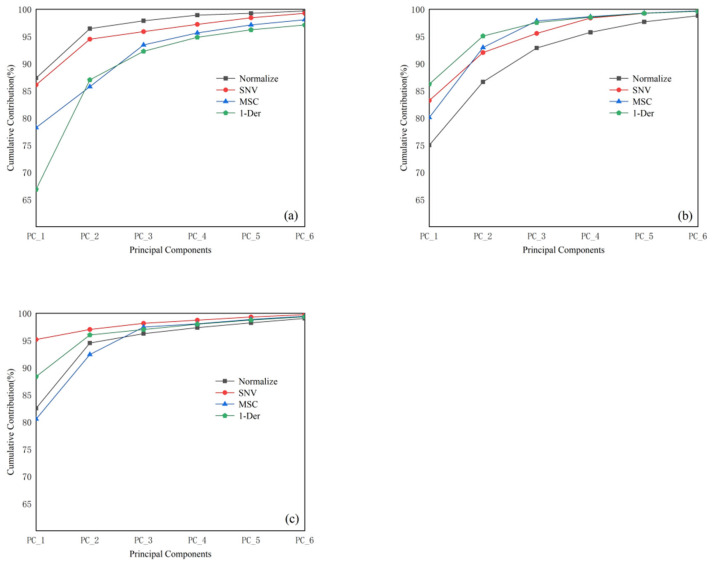
Cumulative contribution of the first six principal components of different preprocessing methods of portable NIR spectra. (**a**) *T. grandis* kernels with shells, (**b**) *T. grandis* kernels without shells, and (**c**) *T. grandis* kernel granules.

PCA was applied to reduce the dimensionality of the spectral data under different states; the results are shown in Figure 3. For *T. grandis* kernels with shells, the first principal component (PC1) after “Normalize” preprocessing accounted for 88.64% of the variance, and the cumulative contribution of the first four principal components reached 99.64%. Based on the clustering effect of the principal components, the first four principal component scores after “Normalize” preprocessing were selected for Mahalanobis distance calculation. For *T. grandis* kernels without shells, the cumulative contribution of the principal components was highest after “MSC” preprocessing, with the first four principal components accounting for 99.84% of the variance; therefore, the first four principal component scores after “MSC” preprocessing were selected for Mahalanobis distance calculation. For *T. grandis* kernel granules, the first principal component after “SNV” preprocessing accounted for 95.14% of the variance, and the cumulative contribution of the first four principal components reached 99.97%; thus, the first four principal component scores after “SNV” preprocessing were selected for Mahalanobis distance calculation.

### 3.4. Removal of Outlier Samples with Abnormal Protein Contents in T. grandis Kernels

**Figure 4:**
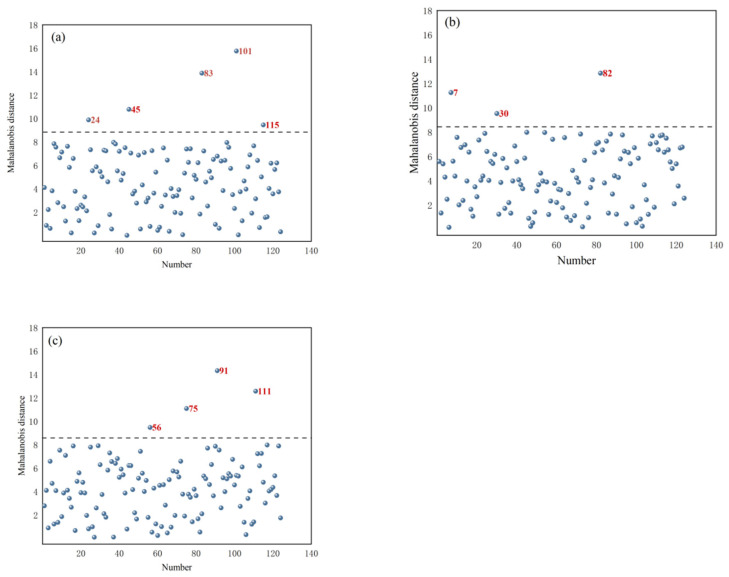
Map of Mahalanobis distance of (**a**) *T. grandis* kernels with shells, (**b**) *T. grandis* kernels without shells, and (**c**) *T. grandis* kernel granules.

Based on the PCA results of the portable NIR spectra of *T. grandis* kernels under different states, the selected principal component score matrix was used to calculate the Mahalanobis distance. The distribution of MD is shown in Figure 4 for *T. grandis* kernels with shells, *T. grandis* kernels without shells, and *T. grandis* kernel granules. In the figures, samples with excessively large MD (marked in red) were identified as outlier samples. From Figure 4a, it can be observed that samples numbered 24, 45, 83, 101, and 115 had excessively large Mahalanobis distance values in the MD distribution of *T. grandis* kernels with shells, resulting in a total of five samples being identified as outlier samples. From Figure 4b, it can be seen that samples numbered 7, 30, and 82 had excessively large MD values in the MD distribution of *T. grandis* kernels without shells, leading to a total of three samples being identified as outlier samples. From Figure 4c, it was evident that samples numbered 66, 75, 91, and 111 had excessively large MD values in the Mahalanobis distance distribution of *T. grandis* kernel granules, resulting in a total of four samples being identified as outlier samples. These outlier samples indicated anomalies in the samples, such as measurement errors, contamination, or other irregularities.

**Figure 5:**
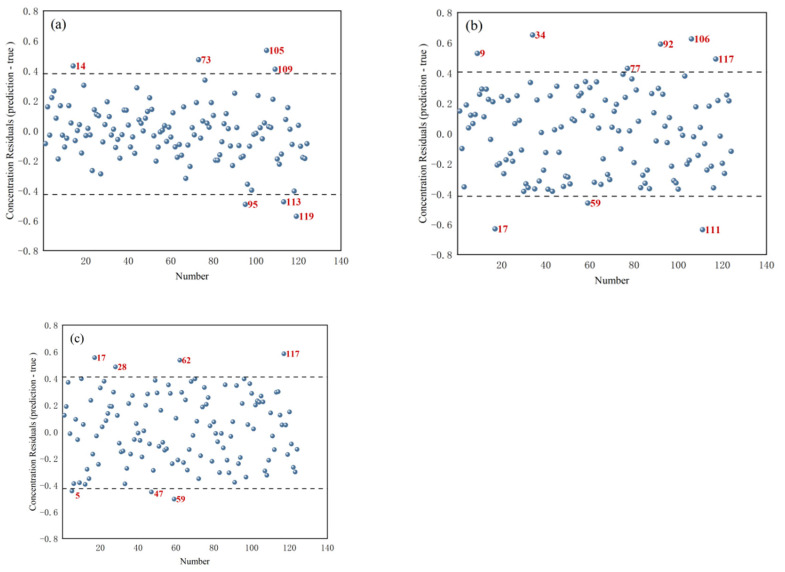
Concentration residual distribution of (**a**) *T. grandis* kernels with shells, (**b**) *T. grandis* kernels without shells, and (**c**) *T. grandis* kernel granules.

Figure 5 shows the concentration residual distribution for *T. grandis* kernels with shells, *T. grandis* kernels without shells, and *T. grandis* kernel granules. In the figures, samples marked in red were identified as outlier samples. For *T. grandis* kernels with shells, samples numbered 14, 73, 95, 105, 113, and 119 (a total of six samples) were removed as outlier samples. For *T. grandis* kernels without shells, samples numbered 9, 17, 34, 59, 77, 92, 106, 111, and 117 (a total of nine samples) were removed as outlier samples. For *T. grandis* kernel granules, samples numbered 5, 17, 28, 47, 59, 62, and 117 (a total of seven samples) were removed as outlier samples. These outliers, identified based on their excessively large absolute concentration residuals (the absolute difference between predicted and measured protein contents exceeded 0.4%), helped to eliminate potential errors caused by measurement inaccuracies, sample contamination, or other anomalies, thereby improving the quality of the data and the robustness of the subsequent modeling.

The outlier samples removed using the PCA-MD and concentration residual methods for the portable NIR spectra of *T. grandis* kernels under different states are listed in Table 2.

### 3.5. Results of Sample Division of T. grandis Kernel Samples

The sample division results for the *T. grandis* kernels after removing outlier samples are shown in Table 3. Due to different outliers being removed under the three different states of *T. grandis* kernels (with shells, without shells, and in the form of granules), the sample division results varied according to state. Overall, the protein content distribution range of the calibration set samples (6.46–12.44%) was wider than that of the prediction set samples (6.52–12.03%) across all three states. This indicates that the sample division was well executed, effectively preventing issues related to model adaptability that could arise if prediction set samples had fallen outside the range of the calibration set.

### 3.6. Establishment and Analysis of a Quantitative Model for Determining Protein Content in T. grandis Kernels

The samples were strategically partitioned into a calibration set (75%) and a prediction set (25%) using the SPXY algorithm after removing outlier samples. This approach optimizes sample selection by maintaining comparable distributions of protein contents in both sets while maximizing spectral variability, thereby ensuring robust model development and reliable performance evaluation. The 3:1 ratio provided sufficient calibration samples for model training while retaining an adequate independent set for prediction. The established quantitative prediction models for determining protein contents in *T. grandis* kernels based on portable NIR spectra under three different states are shown in Table 4. The $R_{c}^{2}$ and $R_{p}^{2}$ values of the protein content quantitative prediction model based on the original portable NIR spectra of *T. grandis* kernels with shells were 0.60 and 0.59, respectively. This model’s predictive performance for determining protein content was relatively poor. The established quantitative prediction models for determining protein content in *T. grandis* kernels exhibited varying degrees of reduced accuracy after preprocessing methods such as 1Der, 2Der, SG, Baseline, and MSC were applied. This was because these preprocessing methods led to the loss of spectral data corresponding to the protein-related bands while reducing noise, resulting in a decline in model accuracy. This highlighted the importance of selecting proper preprocessing methods to preserve essential spectral features while minimizing noise. In contrast, the models for predicting protein content in *T. grandis* kernels established after spectral preprocessing with Normalize, SNV, 1Der+SNV, 2Der+SNV, and SG+SNV showed improved prediction accuracy. This indicated that the preprocessing methods were reasonable and could still enhance the performance of the prediction model. Among these, the model based on PLSR after the combination of the 2Der and SNV (2Der-SNV-PLSR-S) preprocessing methods had the best performance for the portable NIR spectra of *T. grandis* kernels with shells, with $R_{c}^{2}$ and $R_{p}^{2}$ values of 0.69 and 0.67, respectively. This suggests that the high-precision prediction of protein content in *T. grandis* kernels could not be achieved with the shell state.

**Figure 6:**
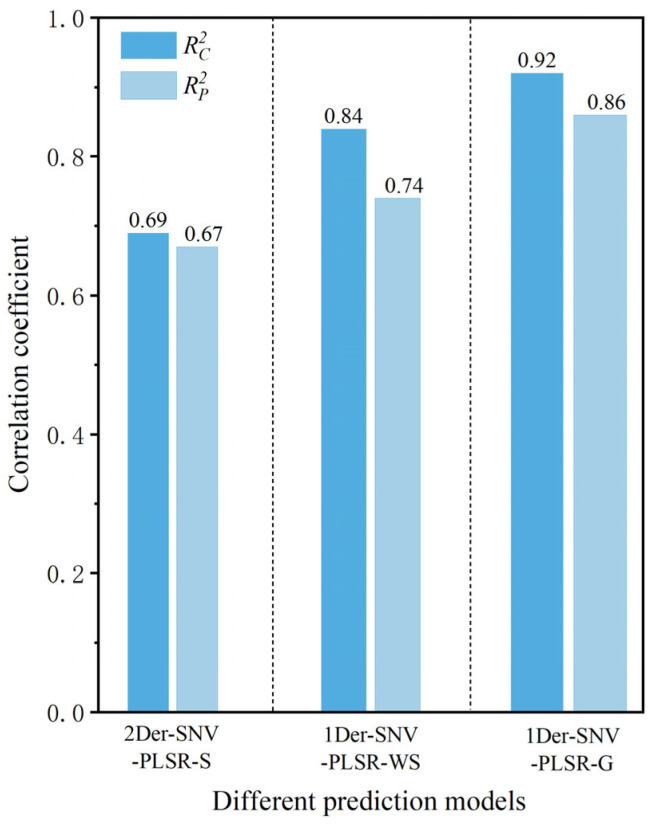
Comparison of different models for predicting protein content in *T. grandis* kernels.

The $R_{c}^{2}$ and $R_{p}^{2}$ values of the protein content quantitative prediction model based on the original portable NIR spectra of *T. grandis* kernels without shells were 0.70 and 0.68, respectively. Compared to the protein quantitative prediction model based on *T. grandis* kernels with shells, the predictive performance of the model based on the portable NIR spectra without shells was significantly improved. Among these, the model based on PLSR after combined preprocessing using 1Der and SNV (1Der-SNV-PLSR-WS) had the best performance, with $R_{c}^{2}$ and $R_{p}^{2}$ values of 0.84 and 0.74, respectively (Figure 6). Compared to the optimal prediction protein model in the shelled state, the $R_{c}^{2}$ and $R_{p}^{2}$ values increased by 21.17% and 10.74%, respectively. It was concluded that the predictive performance of the optimal protein model preprocessed using 1Der-SNV-PLSR-WS, based on deshelled *T. grandis* kernels, was superior to that of the optimal model preprocessed using 2Der-SNV-PLSR-S for kernels in the shelled state. After deshelling, the near-infrared light could directly penetrate the pseudotesta of the kernel, allowing the spectral information of the kernel to be better captured. As a result, the spectral information in the protein-related bands was more complete, leading to a higher accuracy of the established model.

The $R_{c}^{2}$ and $R_{p}^{2}$ values of the protein content quantitative prediction model based on the original portable NIR spectra of *T. grandis* kernel granules were 0.80 and 0.79, respectively. The model’s predictive performance for protein content was the highest among the three states of *T. grandis* kernels. The models established after spectral preprocessing showed an improved prediction accuracy. Among these, the model based on PLSR after combined preprocessing using 1Der and SNV (1Der-SNV-PLSR-G) had the best performance, with $R_{c}^{2}$ and $R_{p}^{2}$ values of 0.92 and 0.86, respectively (Figure 6). Compared to the optimal protein prediction model that was preprocessed using 2Der-SNV-PLSR-S in relation to kernels in the shelled state, the $R_{c}^{2}$ and $R_{p}^{2}$ values increased by 33.43% and 27.98%, respectively. Compared to the optimal protein prediction model that was preprocessed using 1Der-SNV-PLSR-WS in relation to kernels in the unshelled state, the $R_{c}^{2}$ and $R_{p}^{2}$ values increased by 10.11% and 15.56%, respectively. After the *T. grandis* kernels were ground into granules, the portable NIR spectra could capture complete protein-related information. As a result, the predictive accuracy of the model established under this state was the highest.

The optimized protein content quantitative prediction model (1Der-SNV-PLSR-G) based on *T. grandis* kernel granules, with $R_{c}^{2}$ and $R_{p}^{2}$ of 0.92 and 0.86, respectively, indicated that the sample form critically influenced prediction accuracy. However, it required destructive sampling—grinding *T. grandis* kernels into a fine powder, which limited the application of the model for non-destructive detection. This method was ideal for high-precision scenarios such as laboratory research or medical-grade analysis. In contrast, the $R_{c}^{2}$ and $R_{p}^{2}$ of the optimized protein content quantitative prediction model (2Der-SNV-PLSR-S) with shells reached values of 0.69 and 0.67. The RPD and RER were 3.71 (higher than 2.5) and 24.7 (higher than 15), respectively. These data also indicated that the predictive ability of the 2Der-SNV-PLSR-S model had good performance. This model is a non-destructive, suitable, and viable option for large-scale screening and applications where moderate accuracy is acceptable. Its ability to preserve sample integrity made it ideal for rapid field assessments, preliminary quality checks, and high-throughput industrial sorting.

Aulia et al. determined the concentrations of amino acids and crude protein in soybeans using NIRS. They found that sample particle size and the type of spectroscopic instrument significantly influenced the predictive models. Optimizing the grinding and extraction processes increased the $R_{c}^{2}$ value from 0.605 to 0.952 [42]. Similarly, Zhu et al. utilized NIRS combined with PLSR to rapidly and reliably determine the protein content in coffee beans from different regions. The results showed that the OSC-PLSR model performed best for protein content prediction, with an $R_{c}^{2}$ value of 0.982, demonstrating excellent performance metrics [41]. Magwaza et al. developed a rapid and accurate model using NIRS to determine the protein content in sweet potatoes. They established a calibration model using PLSR and achieved the best model performance after 2Der preprocessing. The model demonstrated excellent performance, with an $R_{p}^{2}$ value of 0.98 [43]. The results indicated that NIRS was capable of quickly and accurately predicting the protein content in sweet potatoes. This study confirmed that NIRS combined with PLSR could serve as an alternative method for determining the protein content in nuts. These findings highlight the effectiveness of NIRS and PLSR in accurately predicting biochemical components in agricultural products.

### 3.7. The Predictive Performance of the Optimal Quantitative Model Preprocessed with 1Der-SNV-PLSR-G for Protein Content in T. grandis Kernels

**Figure 7:**
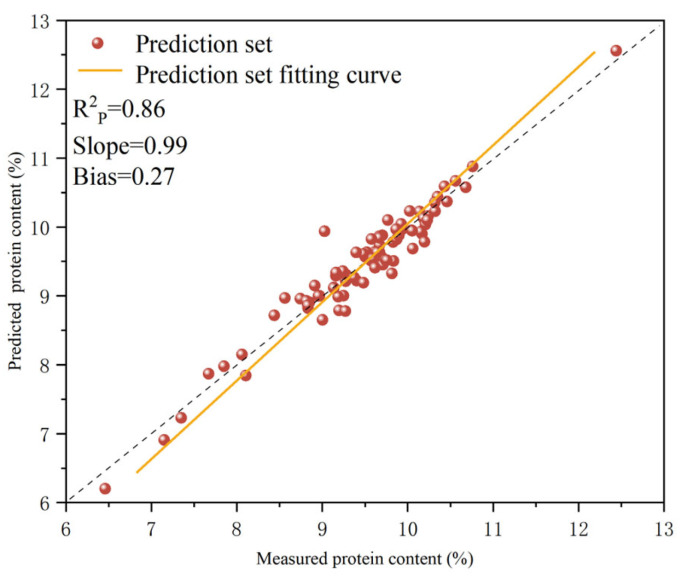
Correlations between measured and predicted protein contents in *T. grandis* kernels.

As indicated above, the optimal quantitative model for determining protein content in *T. grandis* kernels was established using the portable NIR spectra collected from *T. grandis* kernel granules (1Der-SNV-PLSR-G). The samples in the prediction set were input into the optimal model, which was preprocessed using 1Der-SNV-PLSR-G, for protein content prediction; the results are shown in Figure 7. The figure shows that scatter points are distributed on both sides of the fitted line, with no significant outliers, indicating that the model’s predictions were accurate and consistent with the actual measured value. This confirmed the model’s predictive capability. For the optimal quantitative model preprocessed using 1Der-SNV-PLSR-G, the $R_{p}^{2}$ value of the prediction set was 0.86, while the slope and the bias of the model were 0.99 and 0.27, respectively. This demonstrated that the systematic errors were in the controllable range and that the quantitative analysis model established using portable NIR spectra could achieve the precise prediction of protein content in *T. grandis* kernels. The model’s predictive performance met the required standards for practical application. The regression plots reveal a systematic bias (0.27) in the optimal 1Der-SNV-PLSR-G model, particularly at higher protein content ranges. This bias might stem from the uneven distribution of protein in *T. grandis* kernels and the residual matrix effects between the calibration set and the prediction set. Although the current model remains acceptable for screening purposes ($R_{p}^{2}$ = 0.86), the bias can impact absolute quantification in precision-demanding practical applications. The direction of the bias requires incorporating additional spectral preprocessing and expanding the calibration set to better capture the high variability in protein content.

## 4. Conclusions

This study successfully established portable NIRS models for the rapid prediction of protein content in *T. grandis* kernels across three processing states (with shells, shelled, and in the form of granules). The results demonstrated that the optimal model for quantitative protein prediction (1Der-SNV-PLSR-G) based on the portable NIR spectra of *T. grandis* kernel granules was established using PLSR combined with 1Der and SNV preprocessing, with $R_{c}^{2}$ and $R_{p}^{2}$ values of 0.92 and 0.86, respectively. This high precision meets the requirements for practical quality control applications in nut processing. These findings established NIRS as a viable alternative to traditional protein analysis methods for *T. grandis* quality control. The limitations of this study included potential geographical bias due to the exclusive use of samples from the Kuaiji Mountain region and the sole focus on protein content without considering other quality parameters. Future research should expand sample diversity across multiple growing regions and develop multi-parameter prediction models. The methodology provides a foundation for developing rapid, on-site protein detection systems during nut processing and contributes to advancing precision agriculture technologies for specialty crops.

## Figures and Tables

**Table 1:** Statistical analysis of protein content in *T. grandis* kernels.

Component	Sample Size	Maximum/%	Minimum/%	Average/%	Standard Deviation
Protein	124	12.44	6.46	9.64	0.64

**Table 2:** Elimination results of outlier samples for the portable NIR spectra of *T. grandis* kernels under different states.

Sample State	Method	Serial Number
*T. grandis* kernels with shells	PCA-MD	24, 45, 83, 101, 115
	Concentration residual	14, 73, 95, 105, 113, 119
*T. grandis* kernels without shells	PCA-MD	7, 30, 82
	Concentration residual	9, 17, 34, 59, 77, 92, 106, 111, 117
*T. grandis* kernel granules	PCA-MD	66, 75, 91, 111
	Concentration residual	5, 17, 28, 47, 59, 62, 117

**Table 3:** Calibration sets and prediction sets for determining protein contents in *T. grandis* kernels.

Sample State	Calibration Set				Prediction Set			
	Number	Range/%	Mean/%	SD/%	Number	Range/%	Mean/%	SD/%
*T. grandis* kernels with shells	85	6.46–12.44	9.44	0.92	28	6.89–11.83	10.24	0.78
*T. grandis* kernels without shells	84	6.46–12.44	9.48	0.88	28	6.52–11.65	10.12	0.79
*T. grandis* kernel granules	85	6.46–12.44	9.53	0.89	28	6.68–12.03	9.97	0.83

**Table 4:** Modeling results after applying different preprocessing methods.

Sample State	Preprocessing Method	Optimal Number of Latent Variables	Calibration Set		Prediction Set			
			$R_{c}^{2}$	RMSEC	$R_{p}^{2}$	RMSEP	RPD	RER
*T. grandis* kernels with shells	Original	10	0.60	0.29	0.59	0.30	2.60	17.03
	1Der	4	0.54	0.33	0.50	0.36	2.17	13.72
	2Der	4	0.58	0.31	0.57	0.32	2.44	15.44
	SG	6	0.54	0.33	0.51	0.35	2.23	14.11
	Normalize	7	0.63	0.26	0.62	0.27	2.89	18.30
	Baseline	6	0.60	0.30	0.55	0.33	2.36	14.97
	SNV	10	0.65	0.25	0.62	0.27	2.89	18.30
	MSC	8	0.57	0.33	0.53	0.35	2.23	14.11
	1Der+SNV	4	0.66	0.24	0.62	0.26	3.00	19.00
	2Der+SNV	4	0.69	0.20	0.67	0.21	3.71	23.52
	SG+SNV	8	0.66	0.24	0.64	0.25	3.12	19.76
*T. grandis* kernels without shells	Original	10	0.70	0.24	0.68	0.26	3.04	19.73
	1-Der	5	0.81	0.21	0.73	0.29	2.72	17.69
	2-Der	4	0.73	0.24	0.68	0.28	2.82	18.32
	SG	5	0.74	0.23	0.69	0.28	2.82	18.32
	Normalize	7	0.81	0.17	0.78	0.20	3.95	25.65
	Baseline	6	0.74	0.23	0.71	0.26	3.04	19.73
	SNV	10	0.72	0.24	0.69	0.27	2.93	19.00
	MSC	7	0.72	0.24	0.67	0.28	2.82	18.32
	1-Der+SNV	4	0.84	0.19	0.74	0.30	2.63	17.10
	2-Der+SNV	4	0.78	0.21	0.72	0.26	3.04	19.73
	SG+SNV	7	0.72	0.24	0.67	0.28	2.82	18.32
*T. grandis* kernel granules	Original	8	0.80	0.23	0.79	0.25	4.37	28.16
	1-Der	4	0.86	0.26	0.82	0.21	3.32	21.40
	2-Der	4	0.84	0.25	0.74	0.18	3.95	25.48
	SG	6	0.80	0.25	0.86	0.21	4.61	29.72
	Normalize	6	0.83	0.17	0.79	0.24	3.95	25.48
	Baseline	7	0.80	0.23	0.87	0.22	3.46	22.29
	SNV	10	0.85	0.24	0.82	0.17	3.77	24.32
	MSC	7	0.86	0.25	0.82	0.18	4.88	31.47
	1-Der+SNV	7	0.92	0.27	0.86	0.22	4.61	29.72
	2-Der+SNV	5	0.89	0.23	0.72	0.19	3.77	24.32
	SG+SNV	8	0.87	0.25	0.84	0.25	4.37	28.16

## Data Availability

The original contributions presented in the study are included in the article; further inquiries can be directed to the corresponding author.
